# Impact of High-Barrier Packaging Design on Consumer Preference for Not from Concentrated Orange Juice

**DOI:** 10.3390/foods14132356

**Published:** 2025-07-02

**Authors:** Hui Wen, Song Wi, Liya Zhu, Xiaomeng Wu

**Affiliations:** 1 College of Biological Sciences and Technology, Beijing Forestry University, Beijing 100083, China; hhie12304@163.com; 2 College of Food Science and Nutritional Engineering, China Agricultural University, Beijing 100085, China; wisongnongda@cau.edu.cn (S.W.); zlyjulia112358@163.com (L.Z.)

**Keywords:** packaging design, purchase intention, sensory evaluation, high-barrier packaging, NFC orange juice, eye-tracking

## Abstract

This study investigates the influences of the packaging design of not from concentrate (NFC) orange juice on consumer sensory preferences and purchase intention. We conducted a laboratory experiment with eighty-one individuals, combining physiological measurements (eye-tracking to assess attention levels) and sensory evaluation (tasting and rating their overall satisfaction with the taste). Participants evaluated different bottles featuring three design elements (transparency, color, label) but with the same juice inside. In line with the literature on the design of packaging, we show that the position of the transparency, color, and text label consistently alters consumer attention levels and sensory preferences for NFC orange juice. We believe that such findings may guide brand managers and product designers to create more appealing beverage packaging to optimize potential market success.

## 1. Introduction

Consumer attention is inherently drawn to novel and visually salient stimuli, providing actionable insights for packaging design. Key visual elements (e.g., color, transparency, label) significantly influence purchasing decisions by directing attention and shaping expectations [1,2,3]. Peschel et al. [4] demonstrated that larger, more visually salient labels enhance attentional capture and choice likelihood. Critically, these expectations often extend beyond mere visual appeal to encompass anticipated sensory experiences, such as taste, freshness, and texture. This phenomenon is robustly explained by crossmodal correspondence, which posits that associations exist between stimuli across different sensory modalities (e.g., vision and taste) [5]. For instance, specific colors are consistently linked to taste expectations (e.g., red/pink with sweetness, yellow/green with sourness) [6], shapes can imply texture (e.g., angular shapes with bitterness/sourness, rounded shapes with sweetness) [7], and higher levels of packaging transparency are strongly associated with perceptions of freshness and purity [8].

However, packaging design faces unique challenges in certain product categories. Not from concentrate (NFC) orange juice, a premium product requiring low-temperature processing, demands stringent packaging solutions to preserve nutritional integrity and extend shelf life [9,10]. At present, Polyethylene terephthalate (PET) and high-density polyethylene (HDPE) are extensively utilized in the production of bottles for a wide variety of food and beverage products [11,12,13]. The oxygen barrier property of packaging materials is a critical determinant of product quality and shelf life [14,15,16,17]. Several studies have demonstrated that high-barrier plastic packaging materials can significantly reduce oxygen permeability, thereby preserving the original quality of juice products [18,19]. If implemented in juice production, the advantages of NFC juice products, such as flavor retention and nutritional integrity, would be more effectively demonstrated. However, one of the most significant disadvantages of high-barrier packaging is its reduced bottle transparency, which may compromise consumer preference for NFC juice products due to the diminished visual appeal of the contents [20,21]. Given these considerations, the design of high-barrier packaging for NFC juice products is critical to boost its acceptability.

While packaging information was theorized to enhance consumer preference [22,23], studies revealed that most consumers struggle to comprehend textual or ignore textual details due to attention bottlenecks [24,25]. Consequently, understanding consumer decision-making is essential for effective packaging design [26]. As a validated research tool, eye-tracking technology was used to explore how consumers actually process visual information and determine potential attentional focal points based on the information-manufacturing process [27,28]. The data of eye-tracker has the advantages of directness, bidirectional, and naturalness, so it is widely used in the study of consumer behavior [29]. The area tracked by the eye-tracker is called the areas of interest (AOIs) [30]. Fixation time and the number of fixations were the most important investigated eye-tracking parameters indexes in eye-tracking measurement.

This technology effectively reveals how consumers visually engage with elements on packaging. These empirical findings can then be applied to optimize packaging information presentation, thereby improving consumer attention to key nutritional information [31] and fostering healthier food choices. Previous research investigating the sales of Requeijão cheese and similar milk products in comparable ways [32] revealed that all groups were attracted by images on labels related to the product’s origin, suggesting that symbolic visuals may override complex textual information. Building on the aforementioned empirical evidence and the theoretical underpinnings of crossmodal correspondence, we hypothesize that packaging design elements would influence consumer expectations. To test this, we conduct eye-tracking experiments on NFC orange juice packaging, quantitatively analyzing which areas most effectively capture consumer attention in relation to these key visual-sensory cues.

By combining eye-tracking experiments with traditional sensory evaluation, the objective of this study was to assess the association between the design of NFC orange juice high-barrier packaging and purchase intention in consumers’ decision-making process. Eye-tracking data revealed the specific visual elements that most effectively captured attention (measured via fixation time and count within AOIs), identifying attention focal points. Simultaneously, sensory evaluation quantified the influence of these packaging attributes (transparency, color, label design) on taste expectations, freshness perception, and overall liking. It has been demonstrated that the visual attributes of packaging are one of the most crucial sensory indicators significantly influencing a product’s competitive performance on the market shelves [33]. We seek to critically track and record consumers’ eye movements and sensory evaluation scores by providing participants with different packaging of NFC orange juice in a given order. This study would assist designers in proposing more appealing high-barrier packaging to optimize potential market success.

## 2. Materials and Methods

This study employed an eye-tracking experiment and sensory evaluation to record participants’ emotional attitudes towards NFC orange juice with different packages.

### 2.1. Packaging Design

Juice packaging components fall into two categories: visual and textual factors. Color is a significant part of visual effects. Concurrently, textual factors serve as informational vectors designed to facilitate transactional decision-making through product attribute communication [34].

Three variables, including transparency, color, and the text label, are involved in the packaging design. Transparency and color are visual factors that are utilized to elucidate the influence on consumer behavior. Labels, as the textual factors, are applied to opaque bottles to explore whether descriptive information can influence consumer purchasing behavior and the sensory experience of opaque packaging.

The transparency of the packaging container was divided into two groups: transparent and opaque. Therefore, two PET plastic bottles, identical in all aspects except for transparency, were selected for the packaging containers. The opaque bottle was white.

Additionally, the color of the packaging was separated into two groups: one being yellow to present the color of orange juice, and the other being black and white, implicitly associated with artificial flavors and staleness [35]. The packaging patterns were designed in two different hues. The product name (“NFC Orange juice”) and the identical nutrition declaration were included in each design, along with some pictures of related fruits to assist the viewer in connecting and resonating with the juice.

**Figure 1:**
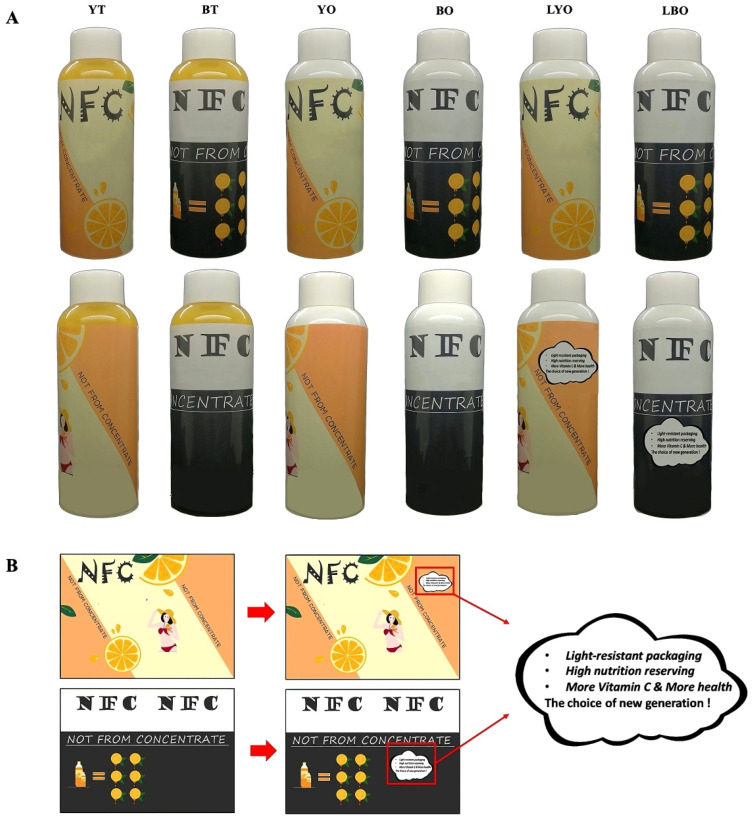
Front and back images of six different packages (**A**) and the text label on them (**B**) were used for the study.

For the opaque bottle, an explanation label was added and placed on the rear to help customers better comprehend the purpose of this type of packaging (Figure 1B).

To be created was six different types of packaging, in accordance with the aforementioned approaches. For the convenience of subsequent descriptions, English names as presented in Table 1 were used to denote the different packages.

### 2.2. Eye-Tracking

#### 2.2.1. Recruitment of Participants

A total of 81 undergraduates aged between 20–26 years at China Agricultural University were invited to take part in the eye-tracking experiment. The ratio of male to female was about 1:3. This demographic represents a significant segment of the juice beverage market, particularly for NFC products, often positioned as premium or healthy options [36,37]. Additionally, the homogeneity of the sample (similar age, educational background, and potentially similar exposure to trends) helps to control for confounding factors when isolating the effects of the specific packaging manipulations under investigation. The participants had no color blindness or color weakness and met the requirements of the eye-tracking experiment. All of them had the consumption habit of purchasing juice products and had received at least one eye-tracking experiment training. The experiment was carried out in the normal daylight room, and the participants sat in front of a telemetric eye tracker with a pupil distance of 60 cm from the display (resolution 1920 × 1080). Before the start of the experiment, gaze calibration was performed using the nine-point calibration method to ensure the accuracy of the data. According to the experimental instructions, the participants gazed at what was shown on the eye-tracker screen. Throughout the experiment, each participant’s gaze behavior while looking at the interface was recorded.

#### 2.2.2. Eye-Tracking Experiment

**Figure 2:**
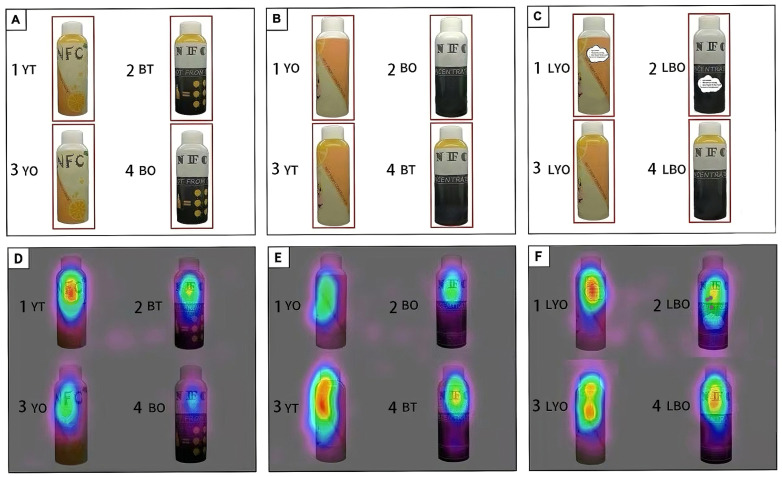
Heatmaps of NFC orange juice front and back packages: red, the highest number of fixations; orange, the second highest number of fixations; yellow, the third highest number of fixations; green, the fourth highest number of fixations; blue, the least number of fixations. (**A**) Front images of packages, (**B**) Back images of packages group 1, (**C**) Back images of packages group 2, (**D**–**F**) Heatmaps of NFC orange juice front and back packages relatively.

The photos of 6 different packages (Table 1) were used in this experiment after background processing. Figure 2A–C is the eye-tracking experiment diagram, in which the part marked by the pink box is the AOIs. The first experimental Figure 2A was the front picture of YT, BT, YO, and BO. The second Figure 2B showed the back of YT, BT, YO, and BO. The third experimental Figure 2C showed the back of YT, BT, LYO, and LBO. Each packaging area was set as the AOI, and fixation time and the number of fixations were automatically recorded by the eye-tracker. At the beginning of each diagram, the instructions appeared on the computer screen, which read, “Please select the sample you would like to purchase from the following four samples.”. After reading the instructions, the participants were shown the experimental diagrams, with each diagram displayed for 20 s.

### 2.3. Sensory Evaluation Experiment

#### 2.3.1. Sample Preparation of NFC Orange Juice

The same batch of frozen NFC orange juice was thawed and then pasteurized (85 °C, 30 s) and filled into the above packaging bottle.

#### 2.3.2. Recruitment of Participants

The same participants formed the sensory panel. All of them had received at least one sensory evaluation training. During the training, evaluators used reference standards (Table 2) to identify and quantify key sensory attributes. Sensory references were developed according to ISO 5492, 2008 [38]. The sensory tests were performed in a standard sensory testing room (ISO 8589, 2007 [39]) according to a completely random block design. The assessors were given drinks as a reward for their experiments in the sensory evaluation room.

#### 2.3.3. Sensory Evaluation Scheme

There was six sample prepared for each assessor (same NFC orange juice in yellow transparent, black and white transparent, yellow opaque, black and white opaque, yellow opaque with label, and black and white opaque with label bottles, respectively). Each sample was presented to the assessor with a three-digit number and placed randomly.

The assessors were given the samples, advised to pour them into small cups, and then asked to rate their preference for color, homogeneity, taste, sweetness, acidity, smoothness, packaging, and overall acceptability of the six samples according to the scoring rules. The sensory evaluation utilized a 10-point graphic interval scale. Table 3 shows the specific scoring rules for each indicator.

### 2.4. Data Analysis

The experiments were conducted in triplicate, and the results were reported as the mean ± standard deviation. Data processing, analysis, and graphic generation were performed using Excel 2010, SPSS 23.0 software, and Origin 2018C. One-way analysis of variance (ANOVA) with Duncan’s test was conducted using IBM SPSS Statistics 25 (SPSS Inc., Chicago, IL, USA) with α = 0.05.

## 3. Results and Discussion

### 3.1. Eye-Tracking Experiment for Different Packaging

Heatmaps developed from the average number of fixations of all participants for the different packages (Figure 2A–C) are shown in Figure 2D, 2E, and 2F, respectively. In the front image of packages, fixations concentrated more on yellow packages (Figure 2(D1,D3)) than black ones (Figure 2(D2,D4)). Relatively high numbers of fixations were found in the transparent packages (Figure 2(D1,D2)) as compared with opaque packages. The yellow-colored transparent package obtained the highest number of fixations. Similarly, in the back image of packages, the yellow-colored transparent package (Figure 2(E4)) obtained more fixations than other AOIs. However, the fixations concentrated on the opaque packages increased when they were attached with text labels that explained the packaging material to participants, especially in yellow packages (Figure 2(F1)). And the fixations are more concentrated on the center point of the labels, whereas it is more distributed across the packaging in the transparent packages (Figure 2(F3)). However, this didn’t seem to have happened in black packages. The different texture of the packaging material didn’t receive more attention from the panelists. On the contrary, panelists were more drawn to the brand name on the packaging.

**Figure 3:**
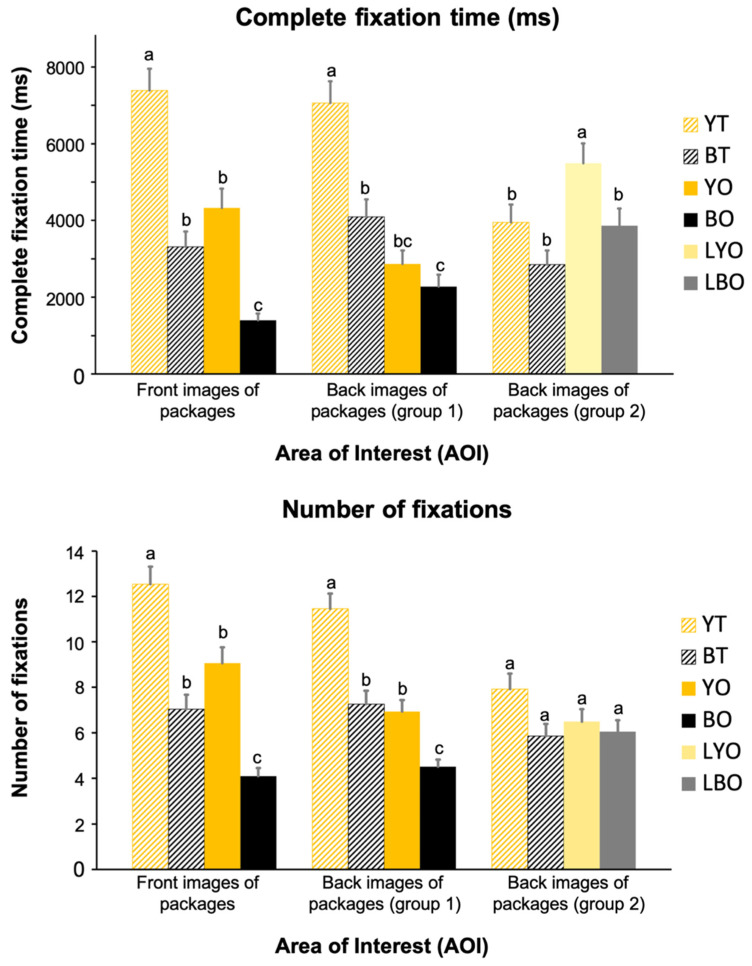
Complete fixation time and number of fixations for all the different packages. A–c means with different superscript letter indicate significant differences (*p* < 0.05) by Friedman’s ranktest.

The fixation points are highly specific to areas and not distributed as zones of focus. As a result, very precise conclusions could not be drawn from the heatmap itself. A quantitative analysis of the complete fixation time (CFT) and the number of fixations (NOF) was conducted to obtain more precise results. The CFT and the NOF mean values of front and back images for the different packages are shown in Figure 3. The front image of the YT package had the highest CFT values compared to those of all other packages (7388.05 vs. 3311.15, 4325.94, and 1402.88 ms, respectively). In the back images of different packages, the YT package also had the highest CFT values compared to those of all other packages (7064.85 vs. 4098.40, 2865.85, and 2279.37 ms, respectively); there were significant increases in CFT for the YT package. This dominance aligns with prior evidence that yellow packaging primes orange juice expectations [40], while transparency enhances perceived freshness [41], collectively amplifying visual salience. Besides, compared with transparent packages, the CFT for the opaque packages with text labels was significantly higher (*p* < 0.05), 5490.83 ms vs. 3953.03 ms in yellow-colored packages, 3868.05 ms vs. 2848.35 ms in black-colored packages. In addition, there were significant (*p* < 0.05) increases in CFT for the LYO package and the LBO package compared with the YO package and the BO package in group 1. It indicated that adding text labels can significantly improve the CFT of the evaluator on the opaque package, which is consistent with the results of the heatmaps.

Similar to CFT, the front image of the YT package had the highest NOF mean values compared to those of all other packages (12.54 vs. 7.05, 9.06, and 4.10, respectively; Figure 3). In the back images of packages, the NOF for the YT package was also significantly higher (*p* < 0.05) than other packages in group 1 (11.46 vs. 7.27, 6.94, and 4.52). But the NOF mean value of the opaque packages with text labels, with no difference (*p* > 0.05) among the four packages in group 2, showed that adding text labels had little influence on the NOF for different packages.

Table 4 and Table 5 showed the generalized linear regression analysis that allowed predicting the overall product acceptability based on the CFT and the NOF of the individual elements in the packaging (transparency, color, label). Two regression models (Fixation time = Intercept + β1*Liking of transparency + β2*Liking of color + β3*Text label + ε; Number of fixations = Intercept + β1*Liking of transparency + β2*Liking of color + β3*Text label + ε) were constructed based on eye-tracking. Regression models confirmed that the color and transparency of packaging were primary drivers of the CFT (color: β = 0.284, 95% CI = [1772.328, 2803.342], *p* < 0.05; transparency: β = 0.263, 95% CI = [1541.728, 2694.440], *p* < 0.05) and the NOF (color: β = 0.311, 95% CI = [2.585, 3.937], *p* < 0.05; transparency: β = 0.241, 95% CI = [1.772, 3.286], *p* < 0.05), likely due to crossmodal correspondences [42]. Notably, text labels increased fixation duration on opaque bottles (b = 1693.772 ms, β = 0.210, 95% CI = [920.509, 2467.035], *p* < 0.05) but not fixation frequency (β = 0.012, 95% CI = [−0.890, 1.136], *p* > 0.05), suggesting labels require cognitive processing but fail to enhance initial visual attraction.

The human brain employs attention mechanisms to filter and prioritize sensory input for higher-order cognitive analysis during stimulus perception [43,44,45]. In consumer contexts, visual attention mechanisms prioritize packaging attributes such as shape and color [46], which critically influence consumer initial evaluations of products displayed on retail shelves [47]. Fixation duration and frequency on visual stimuli serve as validated metrics for assessing consumer cognitive engagement and decision-making processes [48]. Consequently, in this study, fixation time and the number of fixations can be applied to evaluate purchase intention on different packages of NFC orange juice to some extent. The results showed that there were significant differences in the fixation time and number of fixations of consumers on different packages of NFC orange juice, which means that packaging design plays an influential role in consumer purchasing choices.

Results from heatmaps and eye-tracking parameters (Figure 2 and Figure 3) revealed significantly higher fixation time and frequency on yellow packaging compared to black variants. While early studies predominantly examined how altering the product’s intrinsic color modulates sensory perception [49,50,51], recent research emphasizes packaging color’s role in shaping taste expectations and consumption behavior [1,52,53]. For instance, increased yellow saturation on beverage cans heightened perceived “lemony” taste in sensory tests [49]. In this study, yellow-associated packaging likely primed consumer taste expectations, enhancing appetite and product appeal. These results show that reasonable color design has a positive impact on consumer consumption behavior. Notably, opaque packaging forces consumers to rely solely on external color cues, amplifying the packaging color’s influence compared to transparent packaging. This distinction was evident in fixation time disparities: the YT package attracted twice the fixation time of the BT package, while the YO package garnered three times the fixation time of the BO package (Figure 3). This result suggested that the transparency of the package is another important element that may affect the consumers’ fixation on NFC orange juice packages in eye-tracking.

Generalized linear regression models confirmed that transparent packaging elicited more fixations than opaque designs, suggesting higher purchase intention for transparent NFC juice containers (Table 3 and Table 4). Transparent packaging enhances perceived freshness and naturalness in soft drinks by visually exposing the product, which triggers physiological responses like salivation and dopamine-mediated craving [41,54].

In the present study, the text label was added on the opaque bottle to explain to consumers the functions of this kind of packaging. The results from the regression model showed that text labels significantly improved consumers’ fixation time on the YO package (*p* < 0.05), indicating that text labels on packages had an important influence on consumer consumption behavior. People’s familiarity with novel packaging technology was positively correlated with consumer acceptance [55,56]. This may explain consumer preference for LYO with an added label. The study of Deliza et al. [57] also confirmed that labels explaining high-pressure technology increased purchase intent for pressurized pineapple juice. Hence, the communication between product packaging and consumers is important for the promotion of products, especially when we introduce something new. In this study, as a new packaging technology, high-barrier packaging may also need text labels to provide product information to effectively improve the market acceptance due to their opacity.

### 3.2. Sensory Evaluation for NFC Orange Juice in Different Packaging

A traditional sensory evaluation (taste, smell, and visual testing) was conducted to study consumers’ overall liking of the six different packages.

The effect of the type of packaging on perceived juice quality presented significant differences between the NFC orange juice in different packages (Table 6). There were significant differences among different samples in color, homogeneity, and aroma of juice, while there were no significant differences in taste, sweetness, acidity, and smoothness. In terms of color and homogeneity of juice, the score of yellow transparent packaging NFC orange juice was significantly higher than other packaging samples, despite the fact that they are the same juice in different packages. These perceptions can be attributed to that the color and homogeneity of juice are more associated with our visual senses than our taste senses and can easily be interfered with by the packaging of juice rather than actual physicochemical changes in the juice. In this experiment, evaluators were given a whole bottle of NFC orange juice and then poured it into a small paper cup to taste. The presentation of the whole bottle before pouring into cups likely primed expectations about the juice’s appearance and freshness based on the package, which then influenced the subsequent visual assessment of the poured juice’s color and homogeneity. This highlights the powerful role of packaging in shaping expectations of intrinsic product qualities, even when those expectations may not align with the objective sensory experience during consumption for non-visual attributes [22]. The effects of different types of packaging on overall acceptability also presented significant differences between all the NFC orange juice products. The order of overall acceptability was YT (highest) > LYO > BT > YO > LBO > BO (lowest). This was probably because yellow transparent packaging is well known for orange juice [58], the customers usually prefer to choose the thing they are familiar with [59]. However, when customers are informed of the benefits of opaque packaging by the text label on it, the overall acceptability of opaque packaging is improved. The results from traditional sensory evaluation were consistent with the results of the eye-tracking experiment, which further indicated that the eye-tracking experiment was an effective way to study consumer behavior.

**Figure 4:**
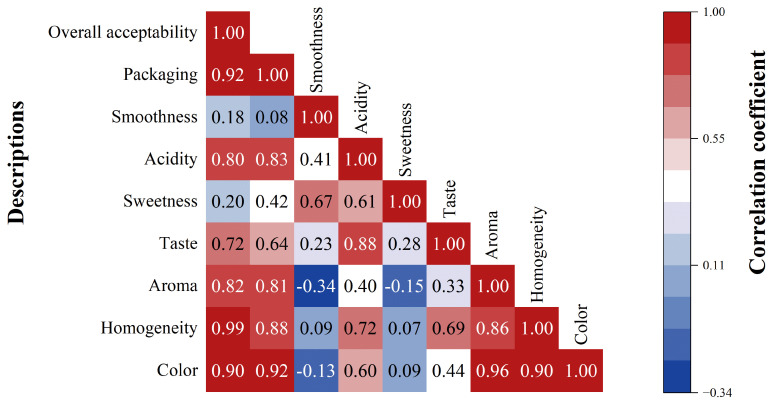
Correlation matrix showing the relationship between sensory evaluation descriptions for NFC orange juice in different packages. The color bar represents the correlation coefficients on a scale from −0.3 to 1, where the blue end denotes the positive correlations, and the yellow end represents the negative correlations.

Figure 4 presents the correlation matrix for sensory evaluation responses of NFC orange juice in different packaging types. Results showed that there were positive correlations between the color, homogeneity, aroma, and overall acceptability of juice and packaging (r > 0.8). Packaging design, as a visual cue, may have a potential impact on taste discrimination [60]. The higher color and aroma scores obtained for preferred packages may be explained by multisensory interactions between packaging design and sensory expectations [61].

Table 7 and Table 8 showed the generalized linear regression analysis that allowed assessing the effects of the individual elements in the packages (transparency, color, label) on the liking of packaging and the overall acceptability sensory scores. Two regression models (Liking of Packaging = Intercept + β1*Transparency of packages + β2*Color of packages + β3*Text label on the packages + ε; Overall acceptability = Intercept + β1*Transparency of packages + β2*Color of packages + β3*Text label on the packages + ε) were constructed based on sensory scores. Table 7 showed that packaging transparency (β = 0.467, 95% CI = [1.258, 2.104], *p* < 0.05), packaging color (β = 0.225, 95% CI = [0.462, 1.156], *p* < 0.05), and text label (β = 0.166, 95% CI = [0.105, 1.087], *p* < 0.05) have a significant influence on packaging score. The average score of transparent packaging was 1.68 points higher than that of opaque packaging. The average score of yellow packaging was 0.81 points higher than that of black packaging. Adding a text label can increase the score by 0.60 points. When it comes to the overall acceptability score, Table 8 showed that the transparency (β = 0.140, 95% CI = [0.123, 0.887], *p* < 0.05) and color (β = 0.114, 95% CI = [0.098, 0.722], *p* < 0.05) of the packaging have a significant influence on the overall acceptability score. The consumers’ overall acceptability of transparent packaging is 0.51 points higher than that of opaque packaging. The overall acceptability score of yellow packaging is 0.41 points higher than that of black packaging. As a result, the scores of liking of packaging and overall acceptability of transparent packaging and yellow packaging were all higher than those of opaque packaging and black packaging. The black packaging and opaque packaging got lower scores due to the mismatch of visual information and the actual products, which reduced the consumer sensory experience of orange juice [62]. In addition, text labels didn’t show any significant influence on the scores of all sensory indicators (*p* > 0.05). This may be because the text label only introduces the uniqueness and nutrition information of the packaging material, and does not involve sensory information.

### 3.3. Limitations

While this study provides valuable insights into the impact of high-barrier packaging design on consumer perception of NFC orange juice, several limitations warrant consideration. Firstly, the research was conducted exclusively within a Chinese consumer demographic (undergraduates aged 20–26). Cultural backgrounds and consumption habits significantly influence food preferences and packaging expectations [63,64,65]. Preferences observed here, yellow transparent packaging may not generalize to consumers in regions with differing juice consumption traditions or color associations [66].

Secondly, the participant sample, while relevant to the target market segment for premium NFC juices [36,37], consisted solely of young, educated individuals. This homogeneity limits the generalizability of findings across broader age groups, income levels, and educational backgrounds, which could exhibit varying levels of familiarity with NFC juice technology and differing sensitivities to packaging attributes like opacity. Furthermore, the notable gender imbalance (male:female ≈ 1:3) may introduce bias, as prior research suggests gender differences in visual attention patterns and sensory sensitivity towards food packaging [67].

## 4. Conclusions

Packaging design influences both NFC orange juice purchase choice and consumer taste experience. The present study examined how color, transparency, and text label influenced consumer eye-tracking responses and sensory preferences toward NFC orange juice. The results demonstrated that color, transparency, and text labels are significant factors in capturing consumer fixation. Yellow packaging and transparent packaging received more fixations, while text labels enhanced fixations on opaque packaging. Sensory evaluation revealed that transparent packaging and yellow packaging scored higher in color, uniformity, liking of packaging, and overall acceptability of NFC orange juice compared to opaque packaging and black packaging. However, text labels did not significantly affect sensory scores. Overall, consumers exhibited greater purchase intention when the packaging color matched the juice’s natural appearance. Even though the transparent bottles are more acceptable, adding labels as part of consumer education can effectively improve the acceptance of opaque bottles. These findings provide important insights for designing high-barrier NFC juice packaging to optimize potential market success. The results underscore the critical importance of utilizing yellow and transparent materials where technically feasible for high-barrier applications to maximize consumer appeal and purchase intention. When opacity is unavoidable due to barrier requirements, strategically placed explanatory labels explaining the technology’s benefits (e.g., freshness preservation, extended shelf life) can enhance engagement and partially offset the visual disadvantage. However, the study’s limitations, particularly its focus on a young Chinese demographic and the potential influence of cultural norms on packaging preferences, necessitate further investigation.

Future studies should be conducted to validate these findings across diverse cultural contexts and broader demographic groups to assess the generalizability of design principles. Additionally, future research should explore the optimization of label content and design to further enhance the appeal and communication efficacy of high-barrier opaque packaging for NFC juices.

## Figures and Tables

**Table 1:** Descriptions of different packaging designs.

Name of Packages	Transparency of Bottle	Color of Bottle	Text Label
YT	Transparent	Yellow	No
BT	Transparent	Black and White	No
YO	Opaque	Yellow	No
BO	Opaque	Black and White	No
LYO	Opaque	Yellow	Yes
LBO	Opaque	Black and White	Yes

**Table 2:** Sensory Reference Standards.

Attribute	Definition	Reference	Intensity (Scale = 0–10)
Sweetness	Basic taste is produced by dilute aqueous solutions of natural or artificial substances such as sucrose or aspartame	5% sucrose solution	5
Acidity	Basic taste is produced by dilute aqueous solutions of most acid substances (e.g., citric acid and tartaric acid)	0.05% citric acid solution	5
Smoothness	Lack of particulate matter	Ultra-homogenized juice (200 MPa)	10

**Table 3:** Assessment criteria for the sensory evaluation questionnaire.

Category	Output	Scale
Color	Dark and poor–Uniform orange	1–10
Homogeneity	Obvious stratification–Very uniform	1–10
Taste	Bad–Good	1–10
Sweetness	Moderate too little–Too much	1–10
Acidity	Moderate too little–Too much	1–10
Smoothness	Very slippery–Very smooth	1–10
Packaging	Very like–Dislike	1–10
Overall acceptability	Very little–Very much	1–10

**Table 4:** Generalized linear regression analysis * predicting the overall product acceptability based on the fixation time of the individual elements in the packages (transparency, color, label).

Variables	Fixation Time Transparency of Packages Color of Packages Text Label			Eye Tracker Setting 1 = Transparent, 0 = Non-Transparent 1 = Yellow, 0 = Black and White 1 = With, 0 = Without		
	Unstandardized Coefficients	Standard Error	Standardized Coefficients	95% Confidence Intervals of Unstandardized Coefficients	t	Pr > |t|
Intercept	1574.342	263.014	-	1058.835, 2089.849	5.986	0.000
Liking of transparency (β1)	2118.084	294.059	0.263	1541.728, 2694.440	7.203	0.000
Liking of color (β2)	2287.835	263.014	0.284	1772.328, 2803.342	8.699	0.000
Text label (β3)	1693.772	394.522	0.210	920.509, 2467.035	4.293	0.000

* Model: fixation time = Intercept + β1*Liking of transparency + β2*Liking of color + β3*Text label + ε. β1, β2, β3 are unstandardized coefficients.

**Table 5:** Generalized linear regression analysis * predicting the overall product acceptability based on the number of fixations of the individual elements in the packages (transparency, color, label).

Variables	Scores of Juice Color Transparency of Packages Color of Packages Text Label			Eye Tracker Setting 1 = Transparent, 0 = Non-Transparent 1 = Yellow, 0 = Black and White 1 = With, 0 = Without		
	Unstandardized Coefficients	Standard Error	Standardized Coefficients	95% Confidence Intervals of Unstandardized Coefficients	t	Pr > |t|
Intercept	4.524	0.345	-	3.848, 5.200	13.116	0.000
Liking of transparency (β1)	2.529	0.386	0.241	1.772, 3.286	6.558	0.000
Liking of color (β2)	3.261	0.345	0.311	2.585, 3.937	9.456	0.000
Text label (β3)	0.123	0.517	0.012	−0.890, 1.136	0.239	0.812

* Model: Number of fixations = Intercept + β1*Liking of transparency + β2*Liking of color + β3*Text label + ε. β1, β2, β3 are unstandardized coefficients.

**Table 6:** Mean values of self-reported responses for the NFC orange juice in different packages.

Evaluation Contents	Package Types					
	YT	BT	YO	BO	LYO	LBO
Color	8.29 ^a^	7.46 ^b^	7.13 ^b^	7.11 ^b^	7.36 ^b^	6.98 ^b^
Homogeneity	8.10 ^a^	7.60 ^b^	7.38 ^b^	7.45 ^b^	7.87 ^ab^	7.40 ^b^
Aroma	7.00 ^a^	6.68 ^a^	6.49 ^a^	6.64 ^a^	6.62 ^a^	6.51 ^b^
Taste	7.21 ^a^	7.07 ^a^	6.98 ^a^	6.91 ^a^	7.28 ^a^	7.17 ^a^
Sweetness	6.39 ^a^	6.56 ^a^	6.53 ^a^	6.21 ^a^	6.47 ^a^	6.38 ^a^
Acidity	6.69 ^a^	6.54 ^a^	6.49 ^a^	6.21 ^a^	6.68 ^a^	6.51 ^a^
Smoothness	7.32 ^a^	7.39 ^a^	7.49 ^a^	7.30 ^a^	7.53 ^a^	7.30 ^a^
Packaging	8.17 ^a^	7.26 ^b^	6.34 ^c^	5.72 ^cd^	7.02 ^bc^	6.23 ^c^
Overall acceptability	7.56 ^a^	7.04 ^b^	6.83 ^b^	6.77 ^b^	7.32 ^ab^	6.79 ^b^

^a–d^ Different superscript letters denote significant differences (*p* < 0.05) between packages. For code information, see Table 1.

**Table 7:** Generalized linear regression analysis * establishing the relationship between liking of packaging and the individual elements in the packages (transparency, color, label) based on sensory scores.

Variables	Liking of Packaging Transparency of Packages Color of Packages Text Label			Sensory Scores 1 = Transparent, 0 = Non-Transparent 1 = Yellow, 0 = Black and White 1 = With, 0 = Without		
	Unstandardized Coefficients	Standard Error	Standardized Coefficients	95% Confidence Intervals of Unstandardized Coefficients	t	Pr > |t|
Intercept	5.628	0.198	-	5.240, 6.016	28.485	0.000
Transparency (β1)	1.681	0.216	0.467	1.258, 2.104	7.766	0.000
Color (β2)	0.809	0.177	0.225	0.462, 1.156	4.575	0.000
Text label (β3)	0.596	0.250	0.166	0.105, 1.087	2.384	0.018

* Model: Liking of Packaging = Intercept + β1*Transparency of packages + β2*Color of packages + β3*Text label on the packages + ε. β1, β2, β3 are unstandardized coefficients.

**Table 8:** Generalized linear regression analysis * establishing the relationship between overall liking and the individual elements in the packages (transparency, color, label) based on sensory scores.

Variables	Overall Acceptability Transparency of Packages Color of Packages Text Label			Sensory Scores 1 = Transparent, 0 = Non-Transparent 1 = Yellow, 0 = Black and White 1 = With, 0 = Without		
	Unstandardized Coefficients	Standard Error	Standardized Coefficients	95% Confidence Intervals of Unstandardized Coefficients	t	Pr > |t|
Intercept	6.593	0.178	-	6.244, 6.942	37.05	0.000
Transparency (β1)	0.505	0.195	0.140	0.123, 0.887	2.592	0.010
Color (β2)	0.41	0.159	0.114	0.098, 0.722	2.573	0.010
Text label (β3)	0.255	0.225	0.071	−0.186, 0.696	1.134	0.257

* Model: Overall acceptability = Intercept + β1*Transparency of packages + β2*Color of packages + β3*Text label on the packages + ε. β1, β2, β3 are unstandardized coefficients.

## Data Availability

The original contributions presented in the study are included in the article; further inquiries can be directed to the corresponding author.
